# Virtual Reality in Training and Assessment Among Clinical Students and Lecturers at a Nigerian University: A Phenomenological Study

**DOI:** 10.2196/75021

**Published:** 2026-01-26

**Authors:** Abiola Olubusola Komolafe, Omotade Adebimpe Ijarotimi, Oluseye Ademola Okunola, Olufemi Mayowa Adetutu, Ayodeji Oludola Oluwatope, Olatunde Abiona, Ojo Melvin Agunbiade, Adeboye Titus Ayinde, Stephen Babatunde Aregbesola, Olawale Babatunde Akinwale, Babatope Ayodeji Kolawole, Lanre Idowu, Alaba Adeyemi Adediwura, Olayinka Donald Otuyemi

**Affiliations:** 1 Department of Nursing Science Faculty of Basic Medical Sciences Obafemi Awolowo University Ile-Ife, Osun Nigeria; 2 Department of Obstetrics, Gynecology and Perinatology Faculty of Clinical Sciences Obafemi Awolowo University Ile-Ife, Osun Nigeria; 3 Department of Sociology and Anthropology Faculty of Social Sciences Obafemi Awolowo University Ile-Ife, Osun Nigeria; 4 Department of Demography and Social Statistics Faculty of Social Sciences Obafemi Awolowo University Ile-Ife, Osun Nigeria; 5 Department of Computer Science and Engineering Faculty of Technology Obafemi Awolowo University Ile-Ife, Osun Nigeria; 6 Department of Computer Information Services College of Arts and Sciences Indiana University Northwest Gary, IN United States; 7 Department of Psychology Faculty of Social Sciences Obafemi Awolowo University Ile-Ife, Osun Nigeria; 8 Department of Oral and Maxillofacial Surgery Faculty of Dentistry Obafemi Awolowo University Ile-Ife, Osun Nigeria; 9 Department of Electronic and Electrical Engineering Faculty of Technology Obafemi Awolowo University Ile-Ife, Osun Nigeria; 10 Department of Medicine Faculty of Clinical Sciences Obafemi Awolowo University Ile-Ife, Osun Nigeria; 11 Department of Educational Technology and Library Studies Faculty of Education Obafemi Awolowo University Ile-Ife, Osun Nigeria; 12 Department of Educational Foundation and Counselling Faculty of Education Obafemi Awolowo University Ile-Ife, Osun Nigeria; 13 Department of Child Dental Health Faculty of Dentistry Obafemi Awolowo University, College of Health Sciences Ile-Ife, Osun Nigeria

**Keywords:** virtual reality, virtual patient, clinical students, health care professionals’ education, digital technology, Nigeria, VR in education, educational technology

## Abstract

**Background:**

Virtual reality (VR) technology is increasingly used in health care professionals’ education as a novel tool for teaching, learning, and assessment.

**Objective:**

This study explored the experiences of clinical students and lecturers with VR for training and assessment at a Nigerian institution. It also explored students’ perceptions of the usefulness of VR in improving their clinical abilities, knowledge retention, engagement, and overall learning experience.

**Methods:**

A qualitative research study was conducted among 24 clinical students and 8 clinical lecturers. A developed Virtual reality model to TRain and Assess Clinical Students (VTRACS) was used to train and assess clinical students using clinical scenarios. Data were collected through 4 focus group discussions conducted among the clinical students and 8 in-depth interviews conducted among the clinical lecturers. Trustworthiness was maintained, and ethical approval for the study was obtained. The focus group discussions and in-depth interviews were audio-recorded, transcribed verbatim, and analyzed using NVivo (version 11; QSR International).

**Results:**

Many of the participants had no previous experience with VR in teaching and learning activities, but judging from their engagement with VTRACS, they defined VR as an alternative learning method (alternative to the traditional physical method). Major themes emerging from the study were expression of excitement, simple and useful innovation, proficiency enhancement, challenges with innovation, and uniformity. The clinical students adjudged VTRACS as an educational supplement with a feeling of unlimited learning access, enhancing clinical abilities while positively impacting their confidence and reducing clinical errors. The participants also described the objectivity and standardization of clinical scenarios as drivers of uniformity in training and assessment of clinical students. The participants were, however, concerned about the loss of empathy with the use of VTRACS, which may negatively impact the affective domain of learning.

**Conclusions:**

The use of VR in the teaching and assessment of clinical students at a Nigerian university is perceived as a complementary method of learning that increases skill acquisition, provides unlimited access to training, and enhances proficiency. While VR is considered to be engaging and beneficial to health care professionals’ education, there is a need for its effective incorporation into clinical courses and mitigation of challenges such as cost and technology to ensure the realization of the full potential of VR in health care professionals’ education.

## Introduction

The challenges posed by the restriction of physical contact during and in the aftermath of the COVID-19 outbreak seem to have suddenly highlighted the need to develop alternative methods of handling teaching and learning activities that require physical contact with a view to attaining desired results [1-3]. In Nigeria, medical training, whose pedagogical design revolves around physical clinical assessment, is no exception.

The increasing adoption of technology, including virtual reality (VR), as an emerging tool in health care professionals’ educational training processes, backed by empirical research, reinforces the credibility of this perspective [4]. VR simulations are engaging, interactive, and realistic, and can improve the learning experience and enable new approaches to the training and assessment of clinical trainees. This technology enables students to participate in complicated clinical scenarios in a controlled and safe setting, allowing for the development of important skills without the hazards associated with real-world patient encounters.

VR has enormous potential in medical education, particularly in resource-limited countries, such as Nigeria. Traditional clinical training approaches frequently encounter obstacles, such as restricted access to clinical facilities, insufficient patient interactions, and inconsistent teaching resources [5,6]. Digital technologies have had a tremendous impact on education, resulting in the implementation of integrated information and communication technology (ICT) initiatives. However, concerns about teaching and learning quality persist, particularly during the COVID-19 pandemic and in other situations that limit physical contact. Many educational institutions have limited experience and digital capacity, which results in learning gaps and disparities in the quality of education and training received by clinical students. This has prompted educational institutions to improve their digital capacity and plan for a successful digital transformation. A nonsystematic literature analysis demonstrated that ICT integration has an impact not only on student achievement but also on school-related factors and stakeholders. Factors associated with digital transformation play an important role in effective and efficient changes in educational institutions [7]. VR presents an opportunity to address these issues by providing standardized, high-fidelity simulations that are accessible to all students regardless of their geographical location.

The adoption of VR in Nigerian health care professionals’ education is still in its nascent stage, and there is a need for empirical research to understand its impact and effectiveness. The use of a developed VR model in clinical students’ instruction and assessment has the potential to alter health care professionals’ education in Nigeria [8]. This study adds to the growing body of literature on the use of VR in education by conducting a contextualized analysis of its impact at a Nigerian university. The findings of this study can help guide future VR implementations in health care professionals’ education and ensure that this technology meets the unique challenges faced by Nigerian clinical students. Understanding the experiences may provide actionable information to the educators, policymakers, and technology developers on future VR implementation in health care professionals’ education to ensure the technology addresses the uniqueness of health care professionals’ education, including the potential benefits and limitations of VR in enhancing health care professionals’ education in Nigeria. This study aimed to explore the experiences of clinical students following their engagement with a developed VR technology software, the Virtual reality model to TRain and Assess Clinical Students (VTRACS), as part of their training and assessment at a Nigerian university. The study hypothesized that the use of the VR model (VTRACS) in training and assessment of clinical students would promote objectivity in clinical assessment, improve students’ clinical abilities, and overall learning experience.

## Methods

### Setting

This study adopted a phenomenological design to explore the experiences of students and faculty members using the VTRACS software in the College of Health Sciences. Phenomenology is especially appropriate for this study because it allowed the researchers to uncover the essence of how participants perceived, interpreted, and made meaning of VR in their educational environment. Phenomenology was applied in the purposeful sampling of participants who had direct experience with VR and in exploring their personal experiences and interpretations of the technology in the results and discussion, rather than focusing on how VR was used. The college comprises the Faculty of Basic Medical Sciences with 2 programs, namely Nursing Science and Medical Rehabilitation, the Faculty of Dentistry with 1 program, and the Faculty of Clinical Science with 1 program, although it has 2 departments: Medicine and Surgery.

The study was conducted using a haptic VR simulation platform, VTRACS, with limited haptic feedback, delivering immersive clinical scenarios through standalone VR headsets (Meta Quest 2). The simulations were designed to replicate common patient care scenarios, such as performing vital signs checks, abdominal examinations (for pregnancy and appendicitis), and conducting tooth extraction, focusing on skill acquisition in nursing, medical, and dental education. The software used a learner-centered approach for the training and assessment of clinical students across the 3 faculties within the College of Health Sciences of the university [8]. Participants interacted with the virtual environment using hand-held controllers, allowing for navigation, action selection, and patient engagement. While the system provided a high level of visual and auditory immersion, haptic feedback was limited to vibrations; no tactile sensations or physical resistance were simulated. Each VR session lasted approximately 20-25 minutes and was conducted in a controlled laboratory environment with technical support available throughout. The content was aligned with curricular objectives and developed to reflect realistic clinical workflows and patient interactions.

### Recruitment Strategy

A total of 32 participants (8 clinical lecturers and 24 clinical students) were recruited for the study. A purposive sampling technique was used to recruit 6 clinical students and 2 clinical lecturers each from Nursing Science, Dentistry, Medicine, and Surgery, who had previously been involved in training and assessment with the VTRACS model. The choice of 6 students from each department was based on the rationale that, unlike quantitative studies, qualitative research focuses on rich, detailed insights from a smaller, carefully selected sample. Research shows that saturation often occurs within 6-12 interviews per homogeneous group, making 6 students per department a reasonable and efficient choice. It also makes data analysis more manageable, especially when using thematic analysis, as was done in this study. Equal representation ensures that comparisons across departments are valid and not skewed by unequal sample sizes. This is crucial when exploring discipline-specific experiences with VR tools in clinical training. Equal numbers help maintain a balanced view of the phenomenon across different clinical disciplines.

The inclusion criteria were being a clinical student and having been trained and assessed in clinical examination with VTRACS, while clinical students who had not been trained or assessed with VTRACS were excluded from the study. The provost of the college and head of departments identified and nominated clinical lecturers with a broad range of disciplinary backgrounds and wide experience who would give diverse insights for the in-depth interviews (IDIs). The students and lecturers had 2 supervised exposures to the VTRACS model before the interviews were conducted.

### Data Collection

VTRACS was used to teach and assess 24 clinical students on clinical scenarios in their field of study. Thereafter, data were collected from the students and the clinical lecturers using an interview guide with 4 open-ended questions (Table 1). Four focus group discussions (FGDs), with an average duration of 45 minutes, involving 6 students from each of the 4 departments, were conducted to explore their experiences with the VTRACS model. The basis for selecting 6 students per department was to ensure equality in the representation of the students. In addition, 8 IDIs lasting an average of 30 minutes were conducted with clinical lecturers from the 4 departments to explore their experiences with the VTRACS model. Qualitative rigor of the study was maintained. To ensure credibility, the research team built rapport with the participants and engaged them in the use of VR before the interviews were conducted. Credibility was also enhanced through member checking, where interview transcripts were given to a few participants who were willing to review their transcripts to confirm accuracy and resonance. Participants’ feedback from the process of member-checking confirmed accuracy and true representation of the interviews. Various strategies contributed to enhancing confirmability in this study. First, data triangulation was ensured by collecting information from multiple sources, such as clinical students in all the faculties in the college and the clinical lecturers. Second, multiple methods, such as FGDs and IDIs, were used to ensure consistency across different datasets. Third, probing followed the flow of the interviews rather than leading, and clarifications were sought where and when necessary. For dependability, documentation of the research process was done transparently through verbatim transcription of all the recorded interviews. All FGDs and IDIs were audio-recorded and transcribed verbatim. An audit trail was maintained, and the researchers kept audio recordings, analysis notes, and coding details. The description of the setting, participants, and context of exploring participants’ experiences with the use of VTRACS allows for the transferability of the study. Qualitative data analysis software, NVivo (version 11; QSR International), was used in organizing data into nodes and themes.

**Table 1:** Sociodemographic characteristics of participants (N=32).

Variable			Faculties (n=8), n (%)		Students (n=24), n (%)
**Age (years)**					
	21-25	—^a^		20 (83.3)	
	26-30	—		4 (16.7)	
	31-35	—		—	
	36-40	—		—	
	41-45	3 (37.5)		—	
	36-40	2 (25)		—	
	≥46	3 (37.5)		—	
**Sex**					
	Male	4 (50)		12 (50)	
	Female	4 (50)		12 (50)	
**Faculty or department**					
	Dentistry	2 (25)		6 (25)	
	Nursing	2		6	
	Medicine	2		6	
	Surgery	2		6	
**Level of study**					
	Lecturing	8 (100)		—	
	400	—		12 (50)	
	500	—		10 (41.7)	
	600	—		2 (8.3)	

^a^Not applicable.

### Ethical Considerations

Ethical approval was obtained from the Health Research Ethics Committee of the Obafemi Awolowo University Teaching Hospitals Complex, Ile-Ife, Nigeria, with protocol number ERC/2022/11/21. The VTRACS project team contacted the potential participants and explained to them the objectives of the study and the purpose of the interviews. Written informed consent was obtained from all the participants, and they were assured of their confidentiality and their right to withdraw from the study at any time without penalty. None of the participants who gave consent to participate in the FGD and IDI withdrew their consent.

## Results

### Characteristics of Participants

A total of 32 clinical students and faculty members participated across the 3 faculties in the College of Health Sciences, as shown in Table 1. Twenty-four clinical students, 6 from each of the 4 departments or faculties, took part in 4 homogeneous FGDs, and 8 clinical lecturers, 2 from each department or faculty, participated in IDIs.

### Themes and Nodes From Participants’ Experience

The results of the study yielded 5 major themes and 13 nodes. The experiences of the students and the lecturers were largely similar, except that lecturers reported more concerns compared to the students, possibly because of generational differences (Gen X and Gen Z) in exposure to and interaction with technology. Textbox 1 provides the themes and their corresponding nodes.

**Themes and nodes from participants’ experience with the Virtual reality model to TRain and Assess Clinical Students (VTRACS).**

**Expression of excitement**

- Novice
- Complementary learning method
- Imagination rather than reality

**Simple and useful innovation**

- Ease of use
- Compatible with the task

**Proficiency enhancement**

- Unlimited learning access
- Confidence
- Fewer medical errors

**Challenges with innovation**

- Limited patient complexity and feedback
- Loss of empathy
- Financial burden of innovation

**Uniformity**

- Standardized clinical scenarios
- Objectivity in clinical assessment

### Theme 1: Expression of Excitement

The introduction of VTRACS for learning by clinical students prepared them for real-world situations, allowing learning and continuous assessment in furtherance of academic excellence and technical expertise. This theme, “expression of excitement,” emerged from the clinical students’ description of their experience with VTRACS as being novice, as an alternative learning method, and as imagination rather than reality. Many of the students and clinical lecturers did not have previous experience with VR for teaching and learning activities, but based their understanding on their interaction with VTRACS. The participants said:

I haven’t used it (VR) before…

VTRACS DENTISTRY FGD 1

Prior to this, I’ve not had…I’ve only heard about it, I’ve not taken part in it.

VTRACS IDI MEDICINE 2

I have not heard about it before…I have not had any experience about it before

VTRACS IDI NURSING 2

I’ve had contact…using VR, but not really in terms of education…education, because there are so many games that require the use of VR and all that…

VTRACS IDI DENTISTRY 2

The definition of VR varies due to the differences in the experiences of the participants with VR. However, a common consensus in the definition of VR is its recognition as an alternative learning method. The participants said:

Virtual reality is more or less an alternative to physical practical where you still have the real-life experience scenario at least to a large extent, and you are able to carry out procedures to an extent like the way you would physically.

VTRACS NURSING FGD 3

To the best of my knowledge, by virtual reality, it’s just like a form of alternative to the usual traditional way of learning…well, I do not really have the details of how it works. I know it has to do with technological modifications

VTRACS IDI MEDICINE 2

The responses from participants in the FGDs and IDIs describe VTRACS as a virtual tool that enhances learning for users who may not have the opportunity for physical training. While some participants viewed it as an alternative, the overall perspective more accurately reflects VTRACS as a complementary method to the traditional physical methods adopted in teaching, particularly in contexts where hands-on experience with real-life patients is essential and available.

The participants demonstrated unfamiliarity with the usage of VTRACS in their statements, but explained their perception of the versatility of VTRACS in teaching and learning from their first experience with it. The participants described the performance of procedures with VTRACS as imagination rather than reality. The imaginative nature of VTRACS immerses users into a different reality where they can explore different and novel learning components. For instance, the participants said:

Virtual reality is like imagination…someone’s imagination like…defined from what you think the reality will be as the name implies – virtual reality, like it’s an imagination.

VTRACS NURSING FGD 4

It’s a simulation…of the clinical procedure which can be used to teach students or…especially online without having an actual patient there. So, I think it’s a very...it’s a very good initiative.

VTRACS DENTISTRY FGD 2

The participant acknowledged VTRACS as a reliable learning platform in the online environment where face-to-face engagements are not possible. This component of VTRACS coincides with the dire need for a substitute method of learning as a result of the limitations imposed by the pandemic. The statement, “It is a very good initiative,” describes the positive perception of VR as an alternative teaching method among clinical students. The new pedagogical method seen in VTRACS and the use of imagination in the performance of clinical procedures, which stems from hands-on experience with VTRACS, gave participants a feeling of excitement. Although participants explained that it did not feel the same as physical practice, this is a limitation in replicating reality, as the use and adoption of VTRACS may not replace traditional physical training in clinical practice, but serves as an alternative.

It was really exciting, comfortable, and actually interesting.

VTRACS NURSING, FGD 1

I also found the experience interesting and…comfortable

VTRACS SURGERY, FGD 6

….it was fun, although it wasn’t like the physical one.

VTRACS MEDICINE, FGD 4

I had a…I had a nice experience, and it was interesting, very interesting…

VTRACS IDI MEDICINE 1

I mean, the experience is something you want to look toward

VTRACS IDI DENTISTRY 1

Although the participants were excited, there was diversity in excitement. While the majority of both students and lecturers found the experience with VTRACS pleasant, comfortable, and interesting, 2 lecturers expressed unpleasantness in the form of headache and dizziness. One of them said:

It was not that pleasant to me when I placed the… accessories, …the one that was put on the head…(headset) okay, so, when I…when I wore it, I discovered that I was having a kind of headache during the process

VTRACS IDI NURSING 2

This experience of discomfort reinforces the existence of individual differences and health disparities in the use of technologies.

### Theme 2: Simple and Useful Innovation

As revealed by the participants’ testimonies, they experienced VR in various ways. Participants who described their experiences as simple also expressed various uses of VR for games, education, watching films, and video demonstrations. This acquaintance with VR made the learning of VTRACS easier because they had previous knowledge of the functioning of VR technological innovation.

It (VTRACS) picked my interest,….and when there is interest, learning becomes much easier. It was quite easy and very logical and systematic in a way. …it’s very clear, and it’s well arranged and very logical.

VTRACS NURSING, FGD 5

It was easy for me to navigate…. the operations and then the…the keys to use, and the instructions were very easy to apply and very easy to follow…..it was so easy to use for me.

VTRACS SURGERY, FGD 3

It gives room for multiple practice…it’s easier…students are able to practice more.

VTRACS DENTISTRY, FGD 6

However, some of the participants who had no previous experience with VR found VTRACS interesting but complex at first interaction. For example, one participant said:

it was fun, …..although it was difficult to learn at first, but with patience to adapt to a new system. It’s more difficult than physical one. Yeah, maybe because I’ve not tried it so many times, ….if it’s physical, I know where to place…I know how to reach…

VTRACS MEDICINE, FGD 4

Some lecturers expressed difficulty with hand-eye coordination compared to the students. They, however, opined that learning new technology at their age is good for their cognition. In the words of one of them:

They (procedural steps in VTRACS) were logical. The only thing was coordinating between the brain and the eye movement…that took some time, but like I said, practice makes perfect… one will be stimulating his or her brain in doing such things. If there is something you’ve not been doing before, and you start doing …… it will help each participant whether old or young, particularly the older ones like me.

VTRACS IDI SURGERY 1

### Theme 3: Proficiency Enhancement

The theme “proficiency enhancement” was generated from 3 nodes, namely: unlimited learning access, confidence, and fewer medical errors. The nodes evolved from the participants’ responses to the benefits of VTRACS in clinical students’ training. The participants believed it was an avenue for students to learn better and gain more knowledge, even beyond the boundaries of the classroom.

Possible benefits could be that in terms of time flexibility…the students don’t always have to be in the hospital settings…they can learn outside hospital settings…it’s flexible

VTRACS IDI MEDICINE 2

This flexibility, in the opinion of the lecturers, would also give them more time to do other things because they do not have to be physically present to supervise the students at all practice sessions.

The participants’ responses reflected an opportunity for seamless learning, transcending physical barriers and limitations of availability. For instance, one of the participants said:

It (VTRACS) can be used to teach a large class, …. when there is a need for physical distancing, when we have to conduct exams, and we don’t have the patients on ground, we can use the virtual experience to conduct the clinical exams…

VTRACS IDI SURGERY 2

Also, the responses from the participants emphasized the benefits of this technology to society. VTRACS, as a virtual platform for learning, equips learners better and helps them replicate these procedures in real-life situations. This further boosts the confidence of clinical professionals through guidance in performing real-life procedures and reduces cases of medical errors. The urge to be in the field of medicine and clinical sciences may be further encouraged through the adoption and use of this technology, thereby producing more medical professionals to cater to the health needs of an ever-evolving society.

…if students have known so many procedures using VR, and they have understood very well, they’ve learnt it, …it will reduce some errors that are committed in the hospitals

VTRACS IDI NURSING 2

I think if we implement this virtual reality now, it will help both the students and the staff at large…the society at large, …, to be able to …train more students…more doctors will be available

VTRACS SURGERY, FGD 3

### Theme 4: Challenges With Innovation

This theme captures the experiential and financial concerns expressed by participants regarding the implementation and use of VTRACS. As exciting as most of the participants found VTRACS, some participants described VTRACS as unable to replicate the variability and realism of real patient interactions, limiting opportunities for comprehensive clinical feedback. They also expressed concern that the lack of real human engagement reduced their ability to connect emotionally, potentially diminishing the development of empathy. One of the participants said:

…a shortcoming of this virtual reality program is that no two patients are the same, so there is a way you will treat a live patient and that you get a particular type of feedback that will help you become a better doctor, that you won’t get from virtual reality.

VTRACS DENTISTRY, FGD 3

I think the disadvantage is that you will probably be unable to show empathy

VTRACS DENTISTRY FGD 2

The challenges I think will be that…there could be a possibility of losing touch with the empathy that comes with…. …I mean, the affective aspect

VTRACS IDI MEDICINE 2

The participants noted that VTRACS may limit the development of empathy due to the absence of real human interaction. In addition, the lack of comprehensive, responsive feedback in VTRACS may be a potential barrier to professional growth. However, some participants indicated that VTRACS could reduce the anxiety often associated with assessments in traditional physical settings, thereby enhancing performance and proficiency. As one participant explained:

Most times, students make mistakes in the physical method of assessment, not because they don’t know it, but because of anxiety. With this VR, the fact that you are wearing it (headset), and you can’t see the person assessing you, you are comfortable to put what you already know into practice.

VTRACS NURSING, FGD 3

Some of the participants, students and lecturers alike, expressed concerns about the financial implications of acquiring the new technology, which may negatively affect sustainability. One student said:

…that means every student will have it, and obviously, the financial burden will be there.

VTRACS NURSING, FGD 6

One of the lecturers also said:

It might be expensive. I don’t know the cost of this one, but I know with the way things are, it will be.

VTRACS IDI SURGERY 1

Another lecturer said:

I don’t know how much one costs (headset)…in terms of cost containment, I don’t know if it’s that cost-effective in the sense that how many of them will be needed for the number of students that we have.

VTRACS IDI NURSING 2

These concerns were, however, not sufficient to deter them from embracing VTRACS because they perceived the concerns as challenges that could be overcome.

### Theme 5: Uniformity

The theme “uniformity” emerged as a result of participants’ acknowledgment that VTRACS ensures standardized clinical scenarios for all clinical students in teaching and examination, thereby promoting objectivity in clinical assessment. The participants said:

….it (VTRACS) is also standardized…. what the patient is presenting with will be standardized for everybody, not that one patient will have an extremely difficult case, and one person will have an easy case, and they will still be score based on that.

VTRACS DENTISTRY, FGD 3

## Discussion

### Principal Findings

The use of VR in the training and assessment of clinical students presents transformative potential for health care professionals’ education, particularly within the context of a Nigerian university. This qualitative study explored the experiences of clinical students engaged in VR technology (VTRACS) as part of their educational curriculum, highlighting their perceptions of the innovation, usefulness, and the benefits and challenges associated with this approach. Overall, participants expressed excitement about using VTRACS and recognized it as a valuable educational supplement, particularly in contexts where access to traditional hands-on training is constrained. The objectivity in assessment and standardized clinical scenarios was described as a driver of uniformity in clinical training and assessment by the participants. The flexibility and accessibility of VR-based simulation were seen as key strengths, allowing learners to engage with clinical scenarios in a safe, controlled environment, thus enhancing clinical abilities. However, participants also expressed concern about the limited patient complexity and feedback, and the potential loss of empathy with the use of VTRACS.

### Comparison With Previous Work

The findings of this study reveal that VR technology significantly enhances the learning experience of clinical students. The immersive nature of VR allows students to engage in near-realistic clinical scenarios, providing a safe and controlled environment to practice and hone their skills. Richards [9] posited that mixed-reality technologies in health care professionals’ education can boost student engagement by combining virtual and augmented reality imagery. Bridge et al [10] also demonstrated that a VR environment improved mindfulness meditation and allowed pseudo-anonymous interactions with peers and tutors. The students in this study reported an increased level of engagement and motivation, noting that VR simulations are more interactive and enjoyable than traditional learning methods. This aligns with the findings of Lau et al [11], who conducted a study involving nurses as participants and reported that the majority similarly found the immersive VR experience engaging and experienced a strong sense of presence. Both studies underscore the potential of VR to enhance learner motivation through immersive, interactive environments, thereby supporting improved learning outcomes by providing a dynamic and stimulating educational experience.

One of the primary benefits identified by the participants in this study was improvement in clinical skill acquisition and confidence. VR simulations offer repetitive practice opportunities in a safe, controlled environment, without the risk of harm to patients. This repetitive practice is crucial for mastering clinical procedures and building confidence. Supporting this, Kennedy et al [12], who compared traditional and interactive VR instructions for clinical students, found that those who received VR training made 40% fewer errors in a simulated practical setting compared to those who received traditional training, highlighting VR’s effectiveness in reducing human error and enhancing procedural knowledge. Similarly, Lau et al [11] reported that VR can improve nurses’ knowledge and skills, though they emphasized the need for further refinement of VR prototypes to enhance the user experience. In this study, students also noted that the immediate feedback provided by the VR system enabled them to promptly identify and correct mistakes, contributing to more effective and self-directed learning. This finding aligns with a systematic review of 31 studies, which demonstrated that VR-based education significantly improves knowledge and skill outcomes among health care professionals when compared to traditional learning methods, reinforcing VR’s positive impact on clinical competence and learner confidence [13].

The integration of VR into health care professionals’ education has the potential to address accessibility and equity issues. Lakshminarayanan et al [14] affirmed that affordability and integrated experience creators, when combined with augmented reality (AR) or VR, can facilitate low-cost data collection in remote and rural locations. Evidence has shown that the use of VR in health care, particularly in surgical education, is gaining popularity owing to possible advantages such as enhanced preoperative resident exposure and improved patient safety [15]; however, VR may not completely imitate organic tissues or generate realistic scenarios, and persistent challenges, such as inconsistent internet connections and implementation costs, may impede equitable adoption among academic centers. Ibrahim et al [16] investigated the incorporation of equity, diversity, and inclusion (EDI) into clinical simulation instruction for health care trainees and clinicians. They found that the integration of EDI in clinical simulation education improves self-awareness, communication, insight, knowledge, self-efficacy, and competence. The recommendations include a systematic approach to incorporating EDI, creating a digital repository of EDI-focused scenarios, cocreating simulations with individuals from varied backgrounds, ensuring a secure learning environment, and conducting more rigorous research to enhance the science of clinical simulations.

Participants in this study generally perceived VTRACS as a valuable tool for enhancing learning, particularly in contexts where access to traditional hands-on or physical training is limited. In resource-limited settings such as Nigeria, access to high-quality clinical training facilities and real patient interactions can be limited [17]. VR provides a means of bridging this access gap by offering standardized and high-fidelity simulations accessible to all students, regardless of their geographical location or institutional resources. This democratization of training resources can contribute to leveling the playing field and ensuring that all students receive comprehensive and equitable training. Andigema et al [18] emphasized the importance of action and collaboration in implementing and scaling up artificial intelligence advances to improve health outcomes and ensure that African populations receive the health care they deserve. As immersive technologies such as VR continue to evolve, their strategic integration into health care professionals’ education has the potential not only to transform learning experiences but also to drive equitable health care delivery on a global scale.

The ability to engage in safe, repeatable, and immersive clinical simulations was highlighted as a key benefit of VTRACS. While some participants initially described VTRACS as an alternative to conventional training, their overall reflections highlighted important challenges with the innovation, such as limited realism and feedback and a reduced capacity to foster empathy, which are typically addressed more effectively in hands-on, physical settings. These challenges underscore the importance of positioning VTRACS not as an alternative but as a complementary training method. This perspective aligns with evidence from previous studies, which support the use of VR in simulation-based learning primarily as a supplement to traditional physical methods, unless realistic haptic feedback is integrated to replicate tactile experiences [19-21]. For example, a systematic review by Rangarajan et al [19] found that haptic-enabled VR simulations significantly enhanced surgical performance compared to nonhaptic VR, highlighting the critical role of tactile realism in effectively substituting physical training. Although the VR platform used in this study includes limited haptic feedback through controller vibrations, it lacks tactile sensations or physical resistance, which constrains its ability to fully replicate real-world interactions or convey empathy. Despite its strong visual and auditory immersion, this limitation reduces the system’s overall realism.

In addition to pedagogical concerns, this study also identified logistical and technical barriers that could impact the effective implementation of VR tools such as VTRACS. Participants reported challenges, including unfamiliarity with the VR learning format, difficulty operating hand controllers, and occasional physical discomfort. These findings are consistent with those reported by Lau et al [11], who observed similar usability issues in their study. These challenges underscore the need to address both ergonomic design and user onboarding when integrating VR-based training into educational settings.

While virtual and augmented reality technology is gaining traction in higher education due to its immersive learning potential, the high cost of acquiring and maintaining VR hardware and software remains a major constraint, particularly for institutions operating under limited budgets. For instance, Marks and Thomas [22] described a purpose-built VR laboratory that housed 26 Oculus Rift headset units. Over 5 teaching sessions, 4833 students were taught in the laboratory, with notable increases in student engagement, especially in the Faculty of Engineering, and a 250% increase (n=1016) in student enrollment. Although 71.5% (n=211) of the students reported improved learning outcomes, the initiative was supported by subsidized funding, highlighting potential cost barriers to wider adoption. Conversely, the shift toward remote education during the COVID-19 pandemic prompted the exploration of VR as a more scalable and cost-effective alternative. A retrospective case study comparing high-fidelity manikin-based simulation and VR-based learning found that VR was 22% less time-consuming and 40% more cost-efficient, supporting its feasibility as a pedagogical solution in resource-constrained environments [23].

Furthermore, technical issues, such as software glitches and hardware malfunctions, were noted by some students, which can disrupt the learning process and cause frustration. A learning curve was also noted, with some users requiring additional training to engage confidently with the platform. These findings echo previous studies that stress the importance of robust institutional support, reliable technology infrastructure, and user training when integrating VR into educational settings. Collectively, these insights reinforce the idea that while VR can meaningfully augment health care professionals’ education, its role should be carefully framed to complement rather than replace traditional hands-on methods.

Cultural and contextual factors also play crucial roles in the implementation of VR in health care professionals’ education. In the Nigerian context, factors such as technological infrastructure, institutional readiness, and cultural attitudes toward technology adoption can influence the success of VR integration. The COVID-19 pandemic has highlighted the crucial role of educational technology in the global education system. However, probably because of a lack of resources, Nigeria has faced challenges in sustaining educational services, resulting in a shift in the school calendar, longer graduation dates, and lower research output. To maintain sustainability, the Nigerian education system must adopt a pragmatic approach to innovation by using the diffusion of innovation theory. However, many practitioners lack the requisite skills and attitudes, require in-service training, and face challenges in having a methodical approach to purchasing and installing ICT infrastructure [24].

Following the use of the headset, some participants raised the issue of headache and dizziness. VR sickness, also known as visually induced motion sickness, can be described as a physiological, unpleasant experience resulting from a mismatch between users’ vestibular and visual stimuli, which can negatively impact the VR user experience [25]. However, certain factors, such as fear, previous experience of motion sickness, and previous exposure to VR, can also influence participants’ experience of VR sickness [26]. Evidence shows that the number of sessions and the intervals between sessions are important factors in increasing adaptation effects and minimizing motion sickness [27-29]. It is usually advisable to start with short sessions, adjust headset settings for comfort, take breaks, avoid intense content, and consult a doctor if the VR sickness persists.

It is essential to tailor the VR content to reflect specific clinical scenarios and cultural nuances relevant to the Nigerian health care system to ensure its effectiveness and acceptance among students and educators. The positive experiences reported by the students in this study suggest a promising future for the integration of VR into clinical education. To maximize the potential of VR, institutions should consider investing in robust technical infrastructure, providing ongoing training and support for both students and faculty, and continuously evaluating and updating VR content to ensure its relevance and accuracy. This is supported by a study that assessed the educational applications of AR and VR across a variety of educational disciplines, concentrating on their impact on student motivation, learning results, engagement, and overall learning experiences [30]. Through simulations, this study investigated how AR and VR can improve information retention and skill acquisition while also encouraging active learning, collaboration, and critical thinking. The study also examined the potential of AR and VR in remote education and made practical recommendations for instructors to effectively incorporate these technologies into their teaching practices [30].

### Strengths and Limitations

The qualitative research design used in this study was well-suited, as it enabled an in-depth exploration of students’ experiences, perceptions, and contextual interpretations related to the use of VTRACS in their learning processes. One of the primary advantages of qualitative research in educational and media didactic contexts is its ability to capture the complexity and nuance of learner engagement with emerging technologies. Through rich, narrative data, qualitative methods used in this study provide insight into how students interpret the effectiveness, usability, and emotional impact of digital learning tools, which are often not easily measurable through quantitative approaches. This is especially relevant when evaluating immersive technologies such as VR, where user experience, perceived realism, and pedagogical value can vary significantly across individuals and contexts.

However, qualitative approaches also have inherent limitations. First, the findings of this study are based on a relatively smaller, nonrepresentative sample, which limit generalizability. Second, the subjective nature of data collection and analysis introduces potential biases from both participants and researchers, despite efforts to ensure reflexivity and transparency. Third, in the context of media didactics, where technological tools are often applied across diverse educational settings, the findings should be interpreted with caution and viewed as context-dependent rather than universally applicable. Nevertheless, the insights gained from qualitative studies can serve as a foundation for further research and development.

### Future Directions

Future work could build on these findings through mixed methods or quantitative study designs to validate trends observed in the qualitative data. Moreover, the findings can inform the iterative design of educational technologies, training programs for lecturers, and institutional implementation strategies, ensuring that pedagogical innovation remains grounded in the lived experiences of learners.

### Conclusion

The use of VR in the teaching and assessment of clinical students is an emerging development in medical education in Nigeria. The use of VTRACS in the training and assessment of clinical students in a Nigerian university was perceived as a complementary learning method that provides continuous access to training and enhances proficiency. While there are hurdles such as cost and technological issues, students’ overall favorable feedback demonstrates VR’s promise as a valuable tool in health care professionals’ education to promote objectivity and improve clinical abilities. Tackling these problems may unlock the full potential of VR in the health care professionals’ education and the development of competent and confident health care professionals in Nigeria.

## Data Availability

The data supporting the findings of this study are available from the corresponding author upon reasonable request.

## Abbreviations

AR: augmented reality
EDI: equity, diversity, and inclusion
FGD: focus group discussion
ICT: information and communication technology
IDI: in-depth interview
VR: virtual reality
VTRACS: Virtual reality model to TRain and Assess Clinical Students
